# AMPK activation induces mitophagy and promotes mitochondrial fission while activating TBK1 in a PINK1‐Parkin independent manner

**DOI:** 10.1096/fj.201903051R

**Published:** 2020-03-22

**Authors:** Alex P. Seabright, Nicholas H. F. Fine, Jonathan P. Barlow, Samuel O. Lord, Ibrahim Musa, Alexander Gray, Jack A. Bryant, Manuel Banzhaf, Gareth G. Lavery, D. Grahame Hardie, David J. Hodson, Andrew Philp, Yu‐Chiang Lai

**Affiliations:** ^1^ School of Sport, Exercise and Rehabilitation Sciences University of Birmingham Birmingham UK; ^2^ Institute of Metabolism and Systems Research University of Birmingham Birmingham UK; ^3^ Mitochondrial Profiling Centre University of Birmingham Birmingham UK; ^4^ Division of Cell Signalling & Immunology School of Life Sciences University of Dundee Dundee UK; ^5^ Institute of Microbiology and Infection, School of Bioscience University of Birmingham Birmingham UK; ^6^ Centre for Endocrinology, Diabetes and Metabolism Birmingham Health Partners Birmingham UK; ^7^ MRC Versus Arthritis Centre for Musculoskeletal Ageing Research University of Birmingham Birmingham UK; ^8^ Centre of Membrane Proteins and Receptors University of Birmingham Birmingham UK; ^9^ Diabetes & Metabolism Division Garvan Institute of Medical Research Sydney New South Wales Australia; ^10^ St Vincent's Clinical School, UNSW Medicine UNSW Sydney Sydney New South Wales Australia

**Keywords:** endogenous, mitophagy, skeletal muscle, tandem ubiquitin‐binding entity (TUBE), ubiquitin

## Abstract

Mitophagy is a key process regulating mitochondrial quality control. Several mechanisms have been proposed to regulate mitophagy, but these have mostly been studied using stably expressed non‐native proteins in immortalized cell lines. In skeletal muscle, mitophagy and its molecular mechanisms require more thorough investigation. To measure mitophagy directly, we generated a stable skeletal muscle C2C12 cell line, expressing a mitophagy reporter construct (mCherry‐green fluorescence protein‐mtFIS1_101-152_). Here, we report that both carbonyl cyanide m‐chlorophenyl hydrazone (CCCP) treatment and adenosine monophosphate activated protein kinase (AMPK) activation by 991 promote mitochondrial fission via phosphorylation of MFF and induce mitophagy by ~20%. Upon CCCP treatment, but not 991, ubiquitin phosphorylation, a read‐out of PTEN‐induced kinase 1 (PINK1) activity, and Parkin E3 ligase activity toward CDGSH iron sulfur domain 1 (CISD1) were increased. Although the PINK1‐Parkin signaling pathway is active in response to CCCP treatment, we observed no change in markers of mitochondrial protein content. Interestingly, our data shows that TANK‐binding kinase 1 (TBK1) phosphorylation is increased after both CCCP and 991 treatments, suggesting TBK1 activation to be independent of both PINK1 and Parkin. Finally, we confirmed in non‐muscle cell lines that TBK1 phosphorylation occurs in the absence of PINK1 and is regulated by AMPK‐dependent signaling. Thus, AMPK activation promotes mitophagy by enhancing mitochondrial fission (via MFF phosphorylation) and autophagosomal engulfment (via TBK1 activation) in a PINK1‐Parkin independent manner.

**Abbreviations**
ACC: acetyl‐CoA carboxylase
AMP: adenosine monophosphate
AMPK: adenosine monophosphate activated protein kinase
CCCP: carbonyl cyanide m‐chlorophenyl hydrazine
CISD1: CDGSH iron sulfur domain 1
DRP1: dynamin‐related protein 1
GFP: green fluorescence protein
MFF: mitochondrial fission factor
MFN‐1/2: mitofusin‐1/2
mtFIS1_101‐152_
: mitochondrial targeting sequence of FIS1 (amino acids 101‐152)
NDP52: nuclear dot protein 52
OPA1: dynamin‐like 120 kDa protein
OPTN: optineurin
OXPHOS: oxidative phosphorylation
PINK1: PTEN‐induced kinase 1
SQSTM1/p62: sequestosome‐1
TBK1: TANK‐binding kinase 1
Ub: ubiquitin
UBA^UBQLN1^
: his‐halo‐ubiquilin1 UBA domain tetramer
ULK1: unc‐51 like autophagy activating kinase 1

## 1 INTRODUCTION

Mitochondria play an important role in maintaining skeletal muscle function.^1^ The removal of defective mitochondria (known as mitophagy) has been implicated in the sarcopenia of aging muscle.^1^ Despite this, our understanding of the molecular mechanisms that regulate skeletal muscle mitophagy remains in its infancy. The lack of tractable tools to study mitophagy and its signaling events in skeletal muscle have made understanding this process difficult. In order to improve our understanding of mitophagy in skeletal muscle, the development of novel tools is required to dissect this process and its signaling at an endogenous level.

Multiple signaling molecules have been implicated in the control of mitophagy, including PTEN‐induced kinase 1 (PINK1), Parkin, AMP‐activated protein kinase (AMPK), BCL2/adenovirus E1B 19 kDa protein‐interacting protein 3 (BNIP3), and BCL2 Interacting Protein 3 Like (BNIP3L, also known as NIX).^2^, ^3^, ^4^ The PINK1‐Parkin signaling axis is the most studied pathway governing mitophagy. In non‐muscle cell lines, under basal conditions, PINK1 is continuously degraded through the N‐end‐rule pathway.^5^ However, upon loss of mitochondrial membrane potential, for example, following uncoupling with the protonophore carbonyl cyanide m‐chlorophenyl hydrazone (CCCP), PINK1 is stabilized and activated on the outer mitochondrial membrane (OMM).^6^ This facilitates PINK1‐dependent ubiquitin (Ub) phosphorylation at Ser 65^7^, ^8^, ^9^, ^10^ leading to the recruitment of Parkin E3 Ub ligase to the OMM.^6^ Allosteric activation of Parkin through phospho‐Ub binding,^11^ along with PINK1 mediated Parkin phosphorylation,^12^ maximally activates Parkin,^13^ allowing Parkin to ubiquitylate OMM proteins such as mitofusin‐1/2 (MFN‐1/2) and CDGSH iron sulfur domain 1 (CISD1).^14^ Ultimately, ubiquitylation of OMM proteins provides a docking site for activated autophagy receptors, such as optineurin (OPTN), nuclear dot protein 52 (NDP52), and sequestosome‐1 (SQSTM1/p62), to bind, linking ubiquitylated cargo to autophagic membranes.^15^ TANK‐binding kinase 1 (TBK1) is thought to be an important signaling node in this process, activating autophagy receptors that link ubiquitylated cargo to the autophagosome.^16^, ^17^, ^18^, ^19^, ^20^, ^21^ TBK1 is known to be activated following phosphorylation at Ser 172^22^ and recent work has suggested that its activation upon mitochondrial depolarization requires both PINK1 and Parkin.^16^, ^17^, ^23^ Despite these advances, much of the research that underpins our current understanding of PINK1‐Parkin mediated mitophagy has been conducted in mammalian cells that stably express non‐native PINK1 and/ or Parkin.^6^, ^7^, ^9^, ^14^, ^16^, ^19^, ^23^, ^24^, ^25^, ^26^ Furthermore, murine models have shown that basal mitophagy occurs in skeletal muscle even in the absence of PINK1.^27^, ^28^ These studies highlight the importance of studying mitophagy and its signaling events using endogenous proteins.

5'‐AMP‐activated protein kinase (AMPK), a critical sensor of cellular energy status, is suggested to be involved in mitophagy.^2^ AMPK facilitates the clearing of damaged mitochondria by promoting fission through the phosphorylation of mitochondrial fission factor (MFF).^29^, ^30^ This process of fission is required for sequestering damaged mitochondria and is suggested to precede mitophagy.^31^ Furthermore, AMPK is required for acute exercise‐induced mitophagy in mouse skeletal muscle via phosphorylation of unc‐51 like autophagy activating kinase 1 (ULK1), which in turn facilitates lysosomal recruitment to damaged mitochondria.^2^ These studies clearly indicate that in skeletal muscle, AMPK activation promotes mitophagy, possibly by inducing mitochondrial fission and lysosomal recruitment.

The important questions in the field of skeletal muscle mitophagy are: (a) whether the PINK1‐Parkin signaling pathway is functionally active; (b) how AMPK is involved in the regulation of mitophagy; and (c) whether it cooperates with PINK1‐Parkin signaling. Therefore, the objective of this study was to elucidate the endogenous mechanisms of mitophagy in skeletal muscle cells. To provide linkage between mitophagy and its intracellular signaling events, we generated a stable skeletal muscle cell line expressing a mitophagy reporter construct (mCherry‐green fluorescence protein [GFP]‐mtFIS1_101‐152_), also known as “mito‐QC”.^27^, ^32^ Moreover, we employed an Ub pull‐down technique to study endogenous ubiquitylation and demonstrate that the PINK1‐Parkin signaling pathway is functional in skeletal muscle cells. However, our results also suggest that in skeletal muscle AMPK drives mitophagy by enhancing mitochondrial fission (via MFF phosphorylation) and mitochondrial autophagosome engulfment (via TBK1 phosphorylation), in a PINK1‐Parkin independent manner.

## 2 MATERIALS AND METHODS

### 2.1 Cell lines and culture

Mouse skeletal muscle C2C12 myoblast cells were obtained from the American Type Culture Collection (ATCC, Manassas, VA, USA). Wild type and PINK1 knockout (KO) HeLa cells were kindly provided by Professor Richard Youle (Biochemistry Section, NIH, Bethesda, MD, USA). Cells were seeded and cultured in DMEM containing GlutaMAX, 25 of mM glucose, and 1 mM of sodium pyruvate, supplemented with 10% of (v/v) fetal bovine serum (GE Healthcare, Buckinghamshire, UK) and 1% (v/v) of Penicillin‐Streptomycin (10 000 Units/mL‐ug/mL). Myoblasts were differentiated into myotubes at 90% confluency in DMEM supplemented with 2% of horse serum (Sigma‐Aldrich, Cambridgeshire, UK). Cultures were maintained in a humidified incubator at 37°C with an atmosphere of 5% of CO_2_ and 95% of air.

### 2.2 Drug reconstitution and cell treatment

CCCP (Sigma‐Aldrich, Cambridgeshire, UK) and adenosine monophosphate (AMP)–activated protein kinase (AMPK) activator 991 (AOBIOUS, MA, USA) were reconstituted as 10 mM and 20 mM (1000x) stocks in DMSO, respectively. Cells were treated with CCCP and AMPK activator 991 as described in figure legends.

### 2.3 Cell lysis

Cells were lysed in ice‐cold sucrose lysis buffer containing: 250 mM of sucrose, 50 mM of Tris‐base (pH 7.5), 50 mM of sodium fluoride, 10 mM of sodium β‐glycerolphosphate, 5 mM of sodium pyrophosphate, 1 mM of EDTA, 1 mM of EGTA, 1 mM of benzamidine, 1 mM of sodium orthovanodate, 1 x complete Mini EDTA‐free protease inhibitor cocktail, 1% of Triton X‐100, and 100 mM of 2‐chloroacetamide. Cell lysates were centrifuged for 15 minutes at 18 000 *g* (4°C) and the supernatant stored at −80°C before analysis for total protein using the Bradford protein assay (ThermoFisher Scientific, Leicestershire, UK). Protein in each sample was quantified from a standard curve using BSA standards.

### 2.4 Ubiquitin pull‐down

His‐halo‐ubiquilin1 UBA domain tetramer (UBA^UBQLN1^) was expressed in Escherichia coli BL21 cells and purified as described previously.^13^ A 200 μL volume of HaloLink resin (Promega, Hampshire, UK), prewashed with phosphate‐buffered saline (PBS) was incubated with 1 mg of HALO‐UBA^UBQLN1^ TUBE protein in 750 μL of binding buffer: 50 mM of Tris‐HCl pH 7.5, 150 mM of sodium chloride, 0.05% of NP‐40, 1 mM of dithiothreitol (DTT) for 2 hours at 4°C. Conjugated HaloLink resin and UBA^UBQLN1^ TUBE protein was incubated with 500‐1000 µg of protein from lysed cell samples prior to overnight rotation at 4°C. After three washes in sucrose lysis buffer (see “Cell lysis”), the enriched Ub and poly‐Ub chains were eluted with 1x NuPAGE LDS sample buffer (ThermoFisher Scientific, Leicestershire, UK). Samples were left to denature overnight at room temperature in 1.5% of 2‐mercaptoethanol.

### 2.5 Western blot

Cell lysates were prepared in 1x NuPAGE LDS sample buffer containing 2‐mercaptoethanol (final concentration 1.5%) and left to denature overnight at room temperature. Ub pull‐down samples and prepared cell lysates (25‐75 µg of total protein) were loaded into 4%‐12% Bis/Tris precast gels (ThermoFisher Scientific, Leicestershire, UK) prior to sodium dodecyl sulfate–polyacrylamide gel electrophoresis (SDS‐PAGE). Gels were run in 1x MOPS buffer for approximately 80 minutes at 150V. Proteins were transferred onto PVDF membranes (Millipore, Hertfordshire, UK) for 1 hour at 100V. Membranes were blocked in 3% of BSA diluted in Tris‐buffered saline Tween‐20 (TBS‐T): 137 mM of sodium chloride, 20 mM of Tris‐base 7.5 pH, 0.1% of Tween‐20 for 1 hour and incubated overnight at 4°C with the appropriate primary antibody. Primary antibodies were diluted in 3% of BSA made up in TBS‐T (see Supplementary Table 1). Membranes were washed in TBS‐T three times prior to incubation in horse radish peroxidase‐conjugated secondary antibodies (see Supplementary Table 1) at room temperature for 1h. Membranes were washed a further three times in TBS‐T prior to antibody detection using enhanced chemiluminescence horseradish peroxidase substrate detection kit (Millipore, Hertfordshire, UK). Imaging was undertaken using a G:BOX Chemi‐XR5 (Syngene, Cambridgeshire, UK). Quantification was performed using ImageJ/Fiji (NIH, Bethesda, MD, USA).

### 2.6 DNA construct and expression

C2C12 myoblasts stably expressing a functionally inert, tandem mCherry‐GFP tag fused to the mitochondrial targeting sequence of FIS1 were generated using the methods described by Allen et al.^32^ Briefly, cDNA for mCherry, GFP, and residues 101‐152 of mouse FIS1 were cloned into a pBABE.hygro vector kindly provided by Dr Ian Ganley. The construct was co‐transfected into HEK293 FT cells with GAG/POL and VSV‐G expression plasmids (Clontech, Saint‐ Germain‐en‐Laye, France) for retrovirus production using lipofectamine LTX reagent (ThermoFisher Scientific, Leicestershire, UK) in accordance with manufacturer's instructions. Virus was harvested 48 hours after transfection and applied to C2C12 myoblasts in the presence of 10 μg/mL polybrene (Sigma‐Aldrich, Cambridgeshire, UK). Cells were selected with 500 μg/mL of hygromycin (Sigma‐Aldrich, Cambridgeshire, UK). In order to obtain a stable homogenous cell line, infected C2C12 myoblasts were sorted and collected based on their expression of mCherry and GFP using FACSAria (BD Biosciences, Berkshire, UK). Sorted C2C12 myoblasts stably expressing the highest relative mCherry‐GFP‐mtFIS1_101‐152_ signal were reseeded and further expanded for characterization and cryopreservation.

### 2.7 Generation of AMPK α1/α2 deficient HEK293 Flp‐In cells

AMPK α1 and α2 deficient HEK293 Flp‐In cells were generated using the CRISPR‐Cas9 technology, as previously described.^33^ The CRISPR sites were identified using the CRISPR Design Tool (http://tools.genome‐engineering.org). The potential targeting oligonucleotides were chosen and a cloning cacc tag was added, as follows: (a) caccGAGTCTGCGCATGGCGCTGC (targets α1 amino acids 1‐4, exon1); (b) caccGAAGATCGGCCACTACATTC (targets α1 amino acids 23‐29, exon1); 3) caccGAGGCCGCGCGCGCCGAAGA (targets α2 intron and ATG start, exon1); 4) caccGAAGCAGAAGCACGACGGGC (targets α2 intron and ATG start, exon1). These oligonucleotides were annealed to their complements containing the cloning tag aaac, and inserted into the back‐to‐back BbsI restriction sites of pSpCas9(BB)‐2A‐Puro (PX459) and pSpCas9(BB)‐2A‐GFP (PX458). HEK293 Flp‐In cells were transfected with 2.5 μg of plasmid DNA, consisting of equal amounts of GFP vector containing oligonucleotides 1 and 2, and Puro vector containing oligonucleotides 3 and 4, for α1 and α2, respectively. After 24 hours, cells were trypsinized and plated into 150 mm plates containing DMEM medium supplemented with 10% of FBS and 0.4 mg/mL of puromycin. After 2 days, the medium was changed and colonies were allowed to form over the course of 7 days. Individual colonies were harvested and amplified before screening for the presence of AMPK α1/α2 by western blotting.

### 2.8 Mitophagy assay

C2C12 myoblasts stably expressing mCherry‐GFP‐mtFIS1_101‐152_ were seeded on imaging dishes (Ibidi, Gräfelfing, Germany) and treated with either 10 μM of CCCP or 20 μM of AMPK activator 991. Following treatment, cells were washed twice with PBS and fixed in 3.7% of formaldehyde with 200 mM of HEPES (pH 7.0) for 10 minutes. After fixing, cells were washed and incubated for 10 minutes in DMEM supplemented with 10 mM of HEPES (pH 7.0), and then washed with PBS before mounting with Prolong gold mounting solution containing 4′,6‐diamidino‐2‐phenylindole (DAPI; ThermoFisher Scientific, Leicestershire UK). Images were taken using a Crest X‐Light spinning disk system coupled to a Nikon Ti‐E base, 60x/1.4 NA (CFI Plan Apo Lambda) air objective and Photometrics Delta Evolve EM‐CCD. For GFP, excitation was delivered at λ = 458‐482 nm using a Lumencor Spectra X light engine, with emitted signals detected at λ = 500‐550 nm. For mCherry, the wavelengths used for excitation and detection were λ = 563‐587 nm and λ = 602‐662 nm, respectively.

### 2.9 Mitophagy and mitochondrial morphology quantitation

Mean fluorescence intensity of both mCherry and GFP, circularity and Feret's diameter were measured in 25 cells from at least 15 fields of view in each condition using ImageJ/Fiji (NIH, Bethesda, MD, USA). The mCherry/GFP ratio of treated cells were normalized to that of their respective DMSO treated control. The relative increase in the mCherry/GFP ratio provides quantification of mitophagy at a whole cell level. For each cell, mean circularity and Feret's diameter were calculated using measurements made on four different sections of the mitochondrial network.

### 2.10 Respirometry

Cellular bioenergetics was measured in intact attached cells as previously described.^34^ Briefly, C2C12 myotubes grown on XF^e^24 microplates exposed to  ±10 μM of CCCP for 24 hours were washed in Agilent Seahorse XF Base DMEM (Agilent Technologies, Manchester, UK) supplemented with 25 mM of glucose, 1 mM of sodium pyruvate, and 2 mM of L‐Glutamine. C2C12 myotubes incubated in Agilent Seahorse XF Base DMEM were inserted into a Seahorse XF^e^24 extracellular flux analyzer (controlled at 37°C) for a 10‐minute equilibration, and four measurement cycles to record basal cellular respiration. Oligomycin (1 μM), FCCP (3 μM), and a mixture of rotenone (1 μM) plus antimycin A (2 μM) were then added sequentially to establish ADP phosphorylation and proton leak rates of respiration; maximum electron transfer capacity; and residual oxygen consumption, respectively. After each addition of these compounds a further four measurement cycles were recorded. For all respirometry experiments, each measurement cycle consisted of a 1‐minute wait, 2‐minute mix, and 3‐minute measurement period and all data were normalized to total protein as quantified by Bradford protein assay.

### 2.11 Statistical analysis

All statistical analyses were performed using GraphPad Software Inc Prism version 8. For time course and dose‐response experiments, a one‐way analysis of variance (ANOVA) was performed with Dunnett's or Sidak's multiple comparisons test where appropriate. For microscopy and seahorse extracellular analyzer experiments, unpaired *t* tests were performed. Data are presented as mean ± SEM.

## 3 RESULTS

### 3.1 CCCP treatment induces mitophagy and promotes mitochondrial fission in skeletal muscle cells

**Figure 1:**
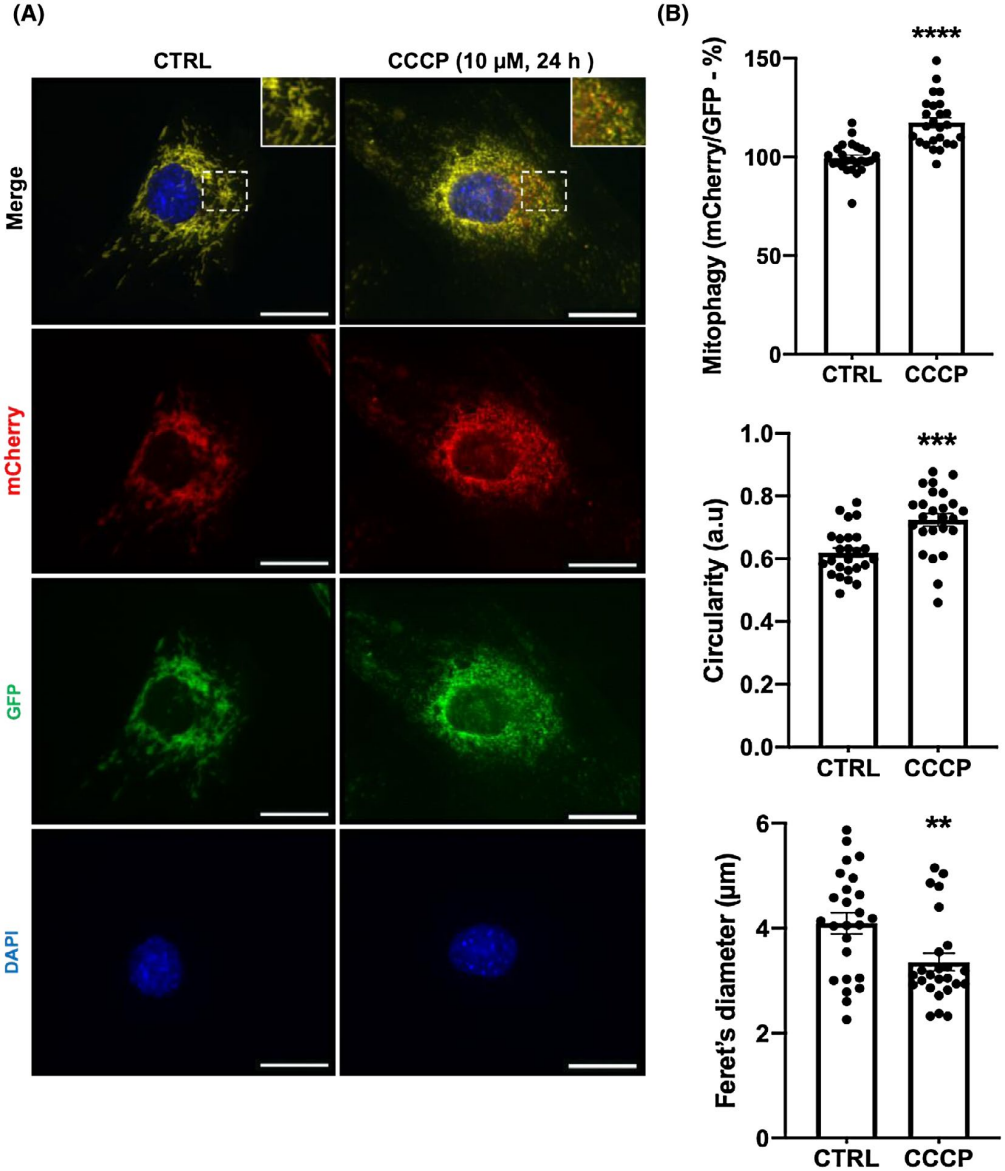
CCCP treatment induces mitophagy and promotes mitochondrial fission in skeletal muscle cells. A, Representative images illustrating CCCP‐stimulated mitophagy and mitochondrial fission in C2C12 myoblasts. C2C12 myoblasts stably expressing mCherry‐GFP‐FIS1_101‐152_ treated with DMSO (0.1%, 24 h) as a vehicle control (CTRL) or CCCP (10 μM, 24 h). Red puncta appearing in the merged image indicate sites of mitophagy. Scale bars = 20 μm. B, Quantification of mitophagy (mCherry/GFP), circularity and Feret's diameter (n = 25 per group). Cells treated as in Figure 1A. Each data point represents one myoblast; mean ± SEM, **P < .01, ***P < .001, ****P < .0001

We generated a mitophagy reporter cell line in C2C12 skeletal muscle cells stably expressing a functionally inert, tandem mCherry‐GFP tag fused to the mitochondrial targeting sequence of the OMM protein FIS1 (mCherry‐GFP‐mtFIS1_101‐152_), as described by Allen et al.^32^ Under steady‐state basal conditions, mitochondria fluoresce both red (mCherry) and green (GFP) making the mitochondrial network appear gold in color when these spectra are merged (Figure 1A; CTRL). However, upon mitophagy, the acidic conditions of the lysosome quench the GFP signal but not mCherry. As a result, punctate structures that fluoresce red only form within the mitochondrial network (Figure 1A; CCCP). The disappearance of GFP signal and the emergence of red puncta indicate sites of ongoing mitophagy (mito‐lysosomes). Upon mitochondrial uncoupling with 10 µM of CCCP treatment for 24 hours, we found that mitophagy increases by 18% in C2C12 myoblasts, as shown by an increase in the ratio of mCherry/GFP (Figure 1B). Moreover, we demonstrate mitochondrial morphology to be altered in response to CCCP treatment, as indicated by the increase in circularity and decline in Feret's diameter (Figure 1B). Together, these data suggest that CCCP treatment promotes fission of the mitochondrial network.

### 3.2 CCCP treatment does not alter mitochondrial protein content despite depolarizing the mitochondrial membrane and impairing mitochondrial respiration

**Figure 2:**
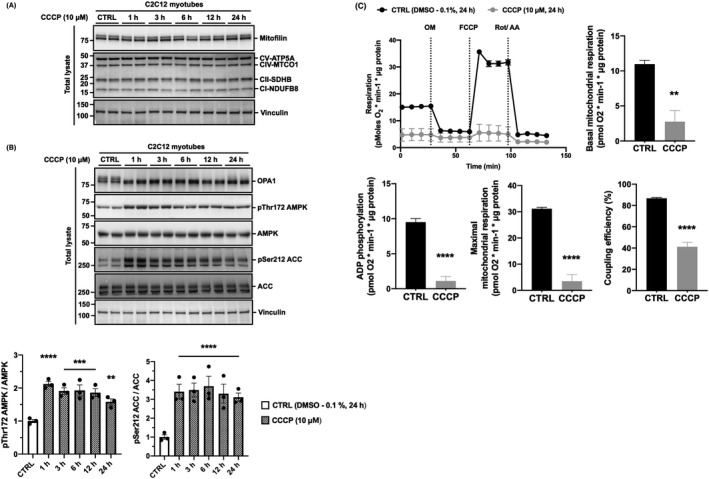
CCCP treatment does not reduce markers of mitochondrial protein content in skeletal muscle cells despite inducing mitochondrial membrane depolarization and impairing mitochondrial respiration. A, Markers of mitochondrial content are unaltered following CCCP treatment for up to 24 h. C2C12 myotubes were treated with DMSO (0.1%, 24 h) as a vehicle control (CTRL) or 10 μM of CCCP for up to 24 h. Total lysates were analyzed by SDS‐PAGE and western blotting with the indicated antibodies. B, CCCP treatment induces mitochondrial membrane depolarization and cellular energy stress in C2C12 myotubes. C2C12 myotubes were treated with DMSO (0.1%, 24 h) as a vehicle control (CTRL) or 10 μM of CCCP for up to 24 h. Total lysates were analyzed by SDS‐PAGE and western blotting with the indicated antibodies. Representative images and quantification of n = 3 independent experiments are shown. Data are expressed as means ± SEM; **P < .01, ***P < .001, ****P < .0001 compared to CTRL. C, Real‐time effects of 24 h CCCP pretreatment on rates of respiration in C2C12 myotubes. C2C12 myotubes were pretreated with DMSO (0.1%, 24 h) as a vehicle control (CTRL) or CCCP (10 μM, 24 h). Oxygen consumption rate was assessed using the Seahorse XFe24 extracellular flux analyzer. Basal mitochondrial respiration was calculated using rates of oxygen consumption measured prior to the addition of oligomycin (OM). ADP phosphorylation respiration was calculated using rates of oxygen consumption sensitive to oligomycin. Maximal mitochondrial respiration was calculated using rates oxygen consumption following uncoupling with carbonyl cyanide‐4‐(trifluoromethoxy) phenylhydrazone (FCCP). Coupling efficiency of oxidative phosphorylation was calculated as the percentage of respiration linked to ATP synthesis. Data were normalized to total protein content and corrected for non‐mitochondrial respiration as assessed by the amount of oxygen consumption remaining after the addition of rotenone (Rot) and antimycin A (AA). Data are expressed as means ± SEM from 1 experimental replicate with four wells per group; **P < .01, ****P < .0001 compared to CTRL

Despite an 18% increase in mitophagy (Figure 1), CCCP had no significant effect on markers of mitochondrial protein content, as monitored by the expression of mitofilin and oxidative phosphorylation (OXPHOS) subunits I‐V (Figure 2A & Supplementary Figure 1). We observed that long isoforms of dynamin‐like 120 kDa protein (OPA1) undergo cleavage after 1 hour of CCCP treatment, indicating depolarization of the mitochondrial membrane potential over the 24 hour time course (Figure 2B). Furthermore, we found increased phosphorylation of AMPK at Thr 172 and of the AMPK target acetyl‐CoA carboxylase (ACC) at Ser 212 after 1 hour of CCCP treatment, indicating increased cellular energy stress (Figure 2B). To confirm deficit in mitochondrial function following prolonged mitochondrial membrane depolarization, we assessed oxygen consumption rate in myotubes pretreated with 24 hour CCCP using extracellular flux analysis (Figure 2C). We show that 24 hour CCCP pretreatment lowers the basal mitochondrial respiration, ADP phosphorylation, uncoupled mitochondrial respiration, and coupling efficiency, indicating impaired mitochondrial respiration (Figure 2C). Altogether, these data demonstrate that in cells not stably expressing non‐native proteins, prolonged mitochondrial membrane depolarization does not reduce mitochondrial protein content in skeletal muscle cells despite impairing mitochondrial respiration.

### 3.3 CCCP treatment induces PINK1 kinase activity and Parkin E3 ligase activity in skeletal muscle cells

**Figure 3:**
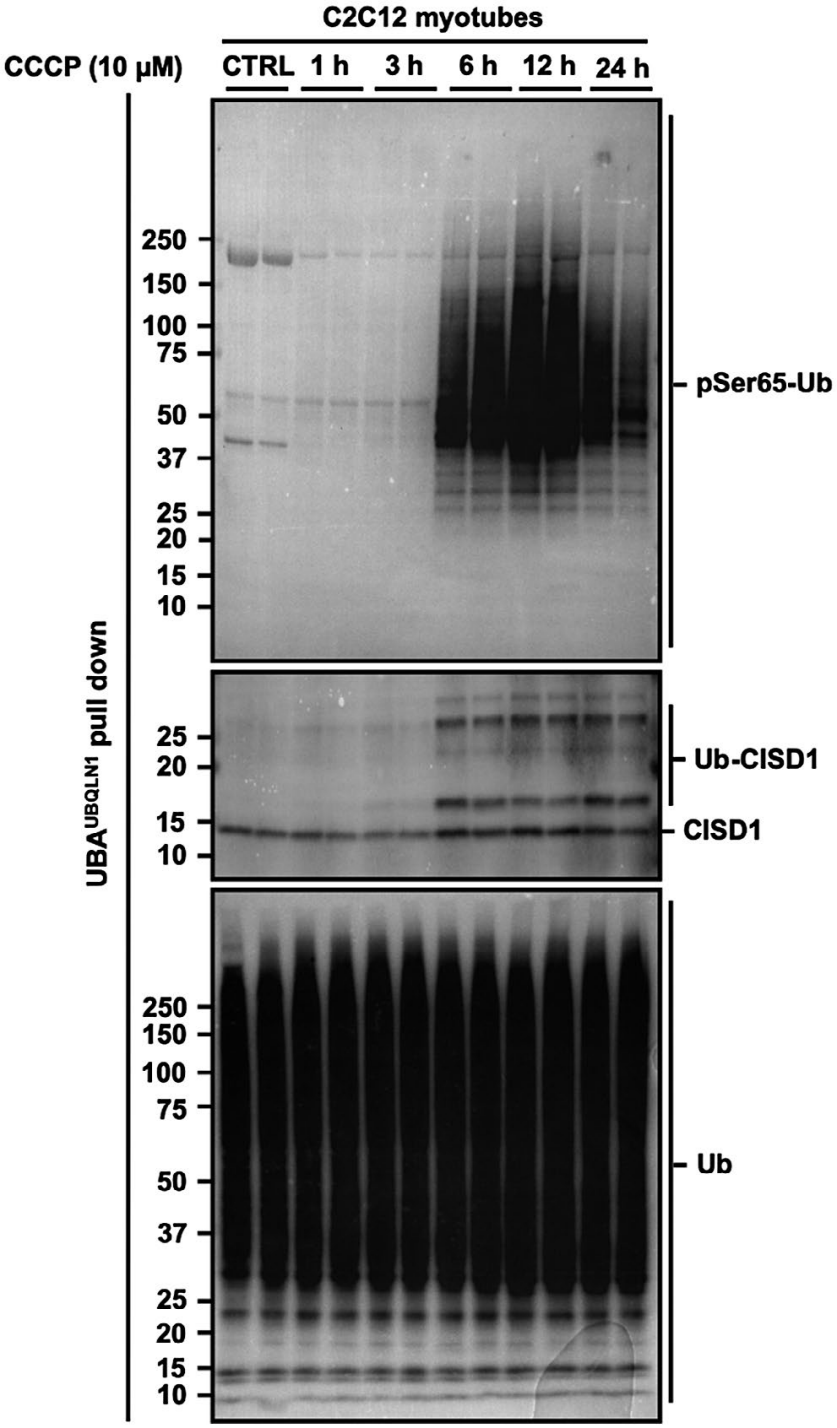
CCCP treatment induces endogenous PINK1 kinase activity and Parkin E3 ligase activity in skeletal muscle cells. C2C12 myotubes were treated with DMSO (0.1%, 24 h) as a vehicle control (CTRL) or 10 μM of CCCP for up to 24 h. Total lysates were incubated with ubiquitin‐binding resins derived from his‐halo‐ubiquilin1 UBA domain tetramer (UBA^UBQLN1^). Captured ubiquitylated proteins were subject to SDS‐PAGE and western blotting with antibodies specific to: phospho‐Ser65 ubiquitin (pSer65 Ub, to determine intracellular PINK1 kinase activity), CDGSH iron sulfur domain 1 (CISD1, to determine intracellular Parkin E3 ligase activity toward its substrate) and total ubiquitin (Ub, to verify ubiquitin enrichment). Representative images of n = 3 independent experiments are shown

Phosphorylation of Ub at Ser 65 and CISD1 ubiquitylation represent intracellular readouts of PINK1 kinase activity^7^, ^8^, ^9^ and Parkin E3 ligase activity,^10^, ^14^ respectively. In order to explore endogenous PINK1 kinase and Parkin E3 ligase activities, we used immobilized haloalkane dehalogenase (HALO)‐ tagged‐UBA^UBQLN1^ tetramers to capture ubiquitylated proteins in whole cell lysates.^35^ Phosphorylation of Ub at Ser 65 and ubiquitylation of CISD1, were increased after 6, 12, and 24 hours of CCCP treatment (Figure 3), consistent with research demonstrating Parkin E3 ligase activity to be regulated by PINK1 activation.^12^ These data demonstrate that endogenous PINK1 kinase and Parkin E3 ligase activities are functional in skeletal muscle cells, although their activation requires prolonged (≥6 hours) CCCP treatment.

### 3.4 CCCP treatment induces phosphorylation of TBK1 in a PINK1‐Parkin independent manner

**Figure 4:**
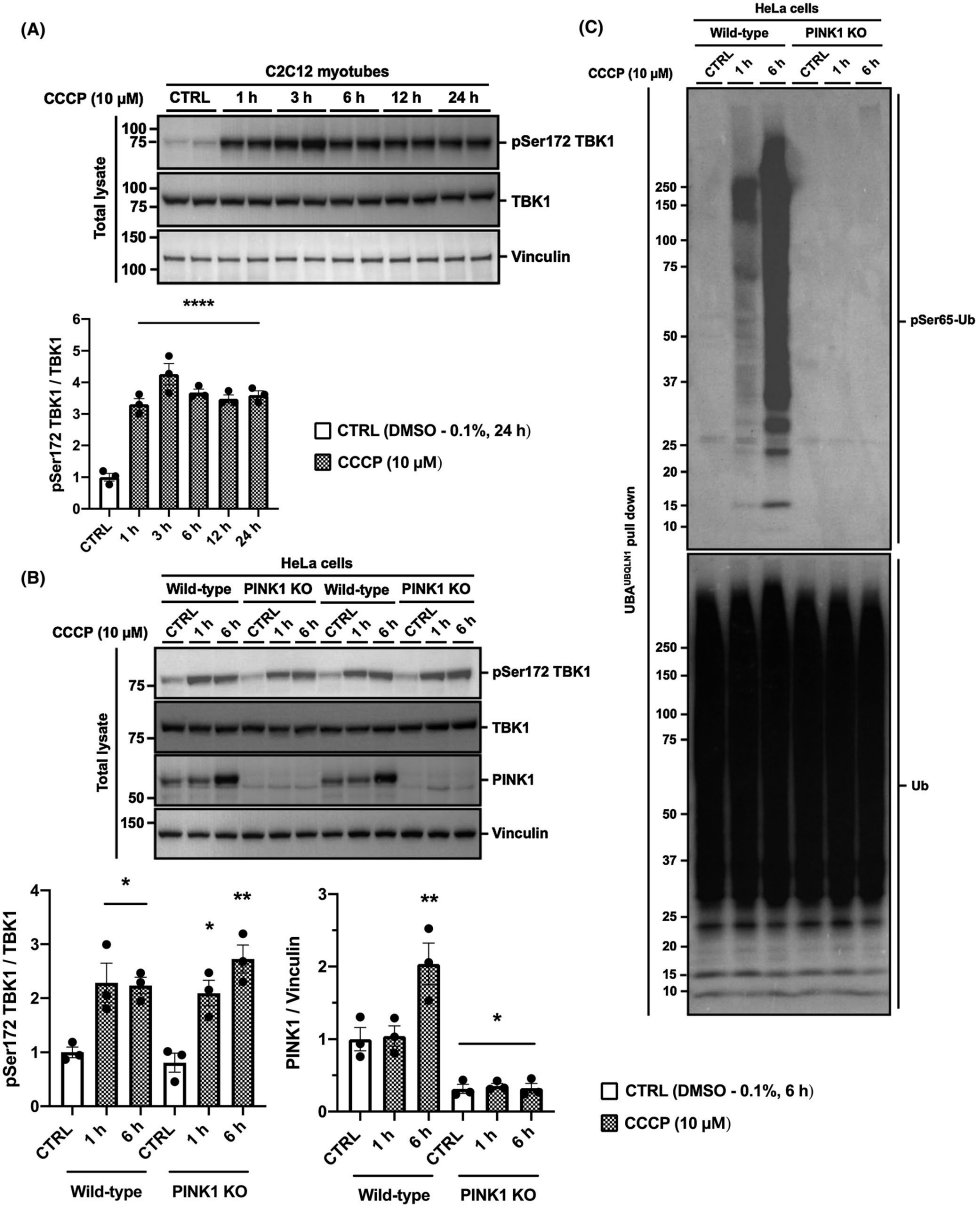
CCCP treatment induces phosphorylation of endogenous TBK1 in a PINK1‐Parkin independent manner. A, TBK1 phosphorylation increases acutely during CCCP treatment. C2C12 myotubes were treated with DMSO (0.1%) as a vehicle control (CTRL) or 10 μM of CCCP for up to 24 h. B, CCCP‐induced TBK1 phosphorylation is independent of PINK1 and Parkin. HeLa wild type and PINK1 knockout (KO) cells were treated with DMSO (0.1%) as a vehicle control (CTRL) or CCCP (10 μM) for 1 and 6 h. Lysates were analyzed by SDS‐PAGE and western blotting with the indicated antibodies. Representative images of n = 3 independent experiments are shown. Data are expressed as means ± SEM; *P < .05, **P < .01, ****P < .0001 compared to CTRL. C, Verification of HeLa PINK1 knockout cells. Ubiquitin was enriched from the total lysates of HeLa wild type and PINK1 KO cells with ubiquitin‐binding resins derived from his‐halo‐ubiquilin1 UBA domain tetramer (UBA^UBQLN1^)

TANK‐binding kinase 1 (TBK1) is thought to be pivotal for the activation of autophagy receptors such as: OPTN, NDP52, and SQSTM1/ p62.^16^, ^17^, ^18^, ^19^, ^20^, ^21^ Recent work has suggested that TBK1 phosphorylation at Ser 172 and its activation,^22^ upon mitochondrial depolarization, requires PINK1‐Parkin signaling.^16^, ^17^ However, we observed increased TBK1 phosphorylation at Ser 172 at all time points from 1 to 24 hours after CCCP treatment (Figure 4A). Given that TBK1 phosphorylation precedes the activation of PINK1 and Parkin (Figure 3), these data suggest that TBK1 activation is independent of PINK1‐Parkin activity in skeletal muscle cells. To verify that phosphorylation endogenous TBK1 in response to CCCP treatment does not require the PINK‐Parkin signaling pathway, we employed PINK1KO HeLa cells. HeLa cells lack Parkin expression,^36^ and so, performing experiments on PINK1 KO HeLa cells would abolish the signaling effects of both PINK1 and Parkin. Strikingly, we still observed TBK1 phosphorylation at Ser 172 in PINK1 KO HeLa cells after 1 and 6 hours of CCCP treatment (Figure 4B), indicating that TBK1 is acutely activated following mitochondrial depolarization in a PINK1‐Parkin independent manner. Knock out (KO) of PINK1 was confirmed via western blotting (Figure 4B). In addition, lack of PINK1 activity was confirmed by an undetectable level of phosphorylated Ub after captured ubiquitylated proteins were subjected to SDS‐PAGE and western blotting (Figure 4C).

### 3.5 AMPK activation by 991 induces mitophagy and promotes mitochondrial fission via phosphorylation of MFF in skeletal muscle cells

**Figure 5:**
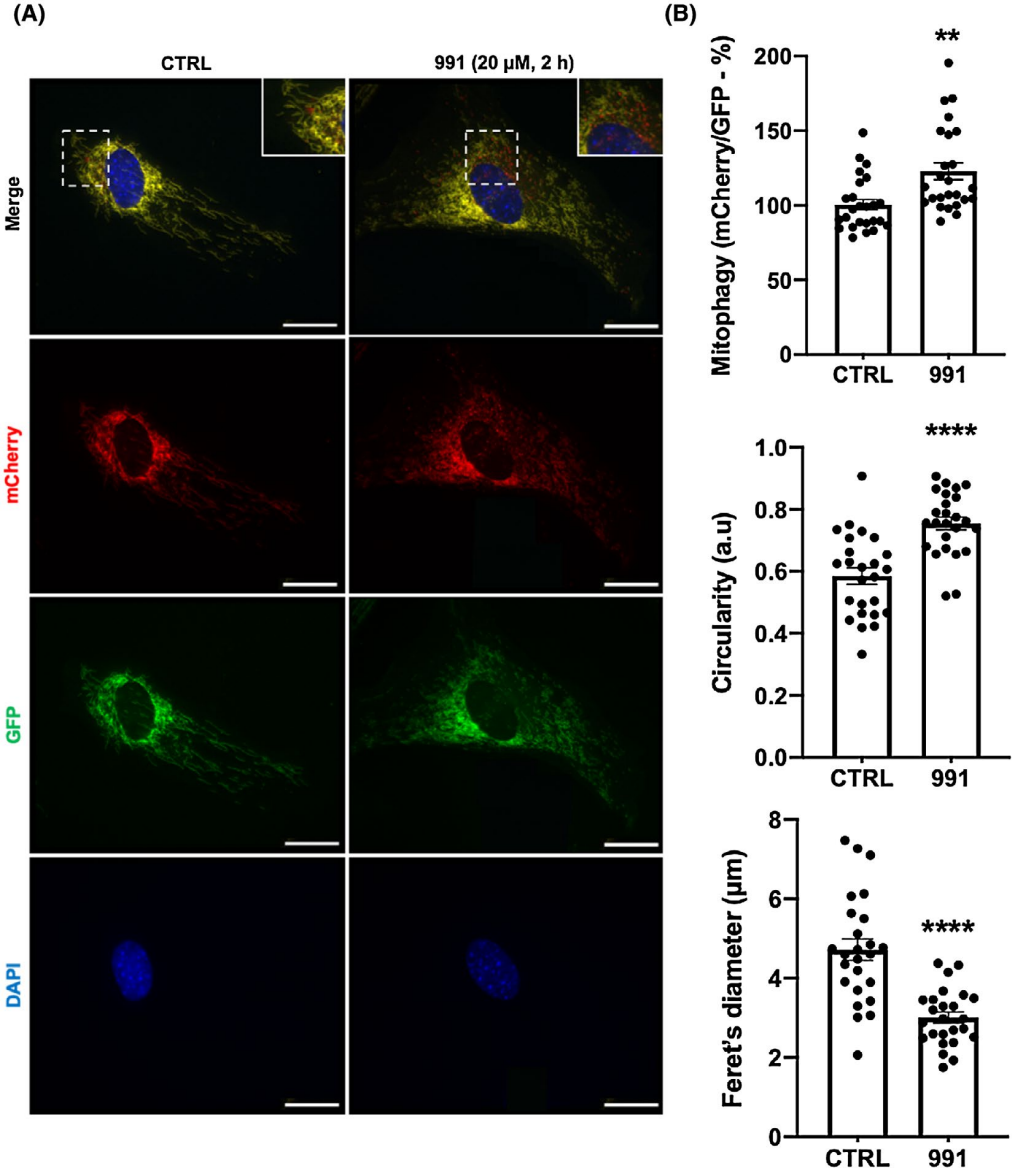
AMPK activation induces mitophagy and promotes mitochondrial fission in skeletal muscle cells. A, Representative images illustrating AMPK activator 991 treatment induces mitophagy and mitochondrial fission in C2C12 myoblasts. C2C12 myoblasts stably expressing mCherry‐GFP‐FIS1_101‐152_ were serum starved for 4 h prior to the treatment of DMSO (0.1%) as a vehicle control (CTRL) or 991 (a specific AMPK activator; 20 μM, 2h). Red puncta appearing in the merged image indicate sites of mitophagy. Scale bars = 20 μm. B, Quantification of mitophagy (mCherry/GFP), circularity and Feret's diameter (n = 25 per group). Cells treated as in Figure 5A. Each data point represents one myoblast; mean ± SEM, **P < .01, ****P < .0001

**Figure 6:**
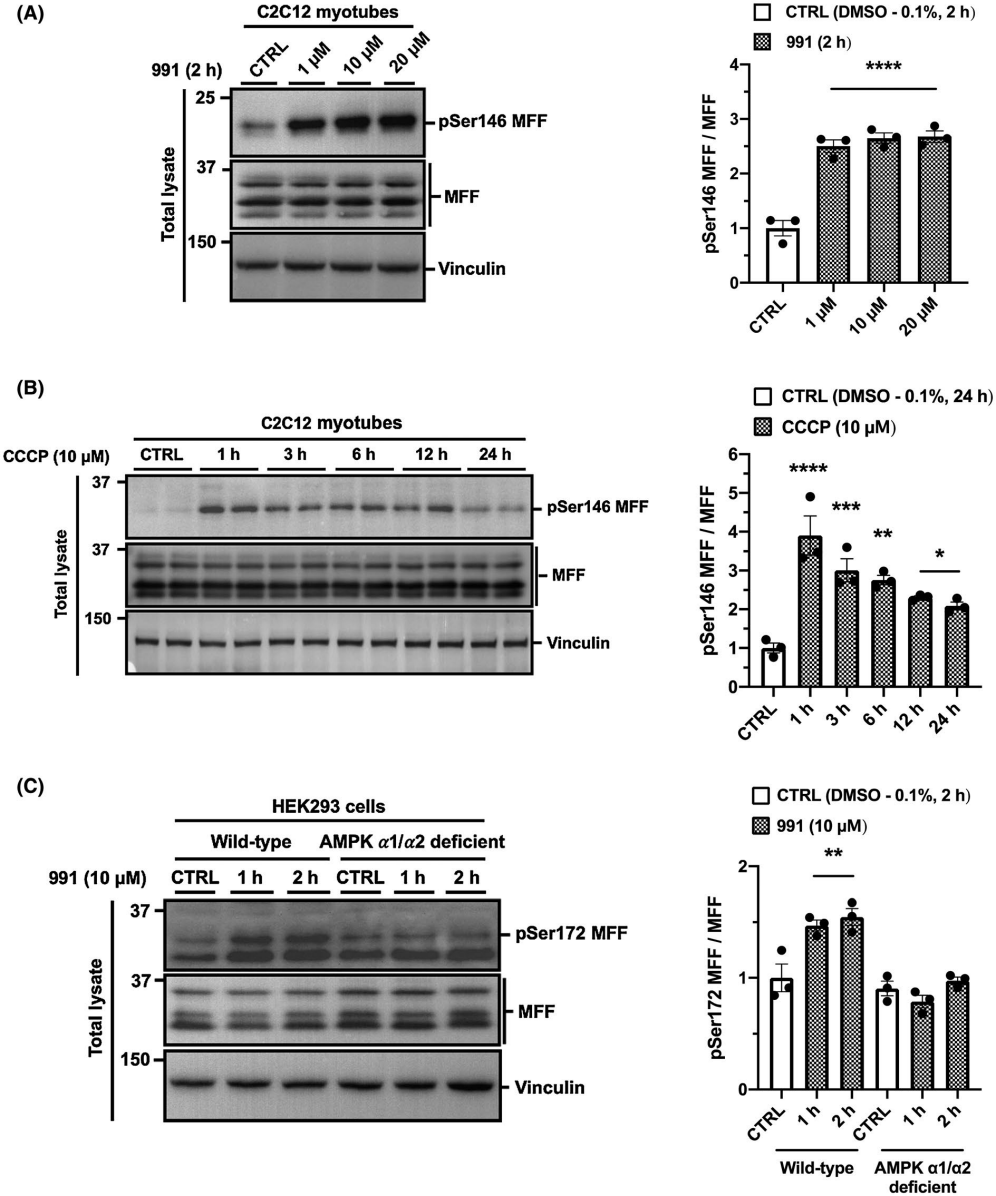
AMPK activation is responsible for MFF phosphorylation at Ser 146 in skeletal muscle cells. A, 991 treatment induces MFF phosphorylation in skeletal muscle cells. C2C12 myotubes were serum starved for 4 h prior to the treatment of DMSO (0.1%) as a vehicle control (CTRL) or AMPK activator 991 at the indicated concentrations for 2 h. B, CCCP treatment induces MFF phosphorylation. C2C12 myotubes were treated with DMSO (0.1%, 24 h) as a vehicle control (CTRL) or 10 μM of CCCP for up to 24 h. C, 991‐induced MFF phosphorylation is diminished in AMPK ⍺1/⍺2 deficient cells. HEK293 wild type and AMPK ⍺1/⍺2 deficient cells were treated with DMSO (0.1%) as a vehicle control (CTRL) or AMPK activator 991 (10 μM) for 1 h and 2 h. Cells were serum starved for 2 h prior to treatment. Total lysates were analyzed by SDS‐PAGE and western blotting with the indicated antibodies. Representative images of n = 3 independent experiments are shown. Data are expressed as means ± SEM; *P < .05, **P < .01, ***P < .001, ****P < .0001 compared to CTRL

Recent work has suggested that mitochondrial fission may spare healthy mitochondria from modification by the PINK1‐Parkin signaling pathway, rather than preparing cells for the clearing of damaged mitochondrial fragments.^31^ Nevertheless, evidence suggests that AMPK‐mediated mitochondrial fission is partly regulated via phosphorylation of MFF.^29^, ^30^ This led us to hypothesize that activation of AMPK may also induce mitophagy following mitochondrial fission. To test this, we used a small‐molecule AMPK activator, 991, to specifically activate AMPK in skeletal muscle cells^37^, ^38^ and found that a 2 h treatment with 991 increases mitophagy by 23% (Figure 5A,B), similar to the effect of CCCP (Figure 1A,B). Moreover, 991 treatment promotes fragmentation of the mitochondrial network (indicated by the increase in circularity and the decline in Feret's diameter) (Figure 5A,B). Consistently, we show a robust increase in MFF phosphorylation at Ser146 (equivalent to Ser 172 in human MFF) in response to 991 (Figure 6A). We also observed increased MFF phosphorylation at Ser 146 in C2C12 myotubes following CCCP treatment from 1 to 24 hours (Figure 6B), in parallel with AMPK activation as indicated by the phosphorylation status of its substrate ACC (Figure 2B). Finally, we employed AMPK α1/α2 deficient HEK293 cells generated using CRISPR‐Cas9 technology (see Section 2). Using this cell line, we confirmed that MFF Ser172 phosphorylation is AMPK dependent (Figure 6C).

### 3.6 AMPK activation by 991 induces endogenous TBK1 activation in a PINK1‐Parkin independent manner in skeletal muscle cells

**Figure 7:**
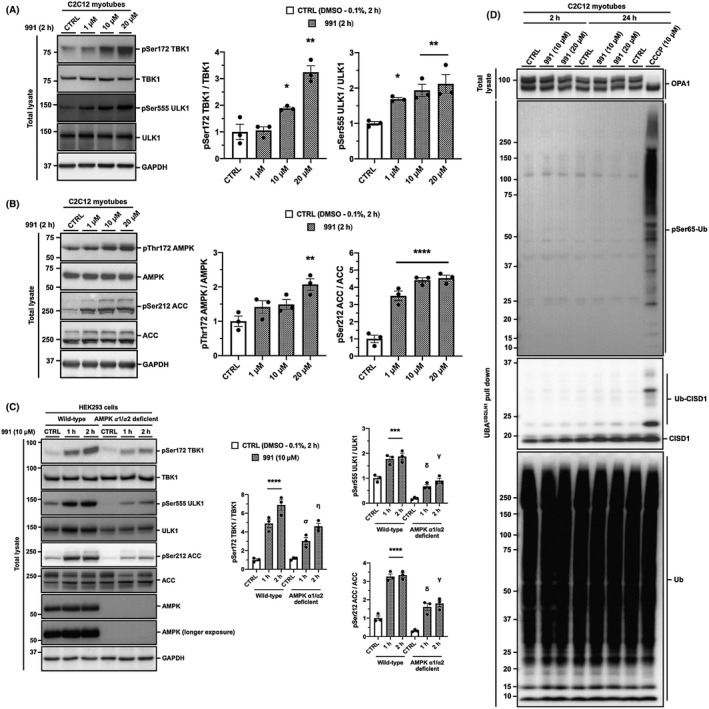
AMPK activation by 991 induces TBK1 activation in a PINK1‐Parkin independent manner. A, 991 treatment induces TBK1 phosphorylation in C2C12 myotubes. C2C12 myotubes were serum starved for 4 h prior to the treatment of DMSO (0.1%) as a vehicle control (CTRL) or AMPK activator 991 at the indicated concentrations for 2 h. B, 991 activates AMPK signaling in C2C12 myotubes. Cells treated as in Figure 5A. C, 991‐induced TBK1 phosphorylation is diminished in AMPK ⍺1/⍺2 deficient cells. HEK293 wild type and AMPK ⍺1/⍺2 deficient cells were treated with DMSO (0.1%) as a vehicle control (CTRL) or AMPK activator 991 (10 μM) for 1 h and 2 h. Cells were serum starved for 2 h prior to treatment. D, AMPK activation does not induce PINK1‐Parkin signaling in skeletal muscle cells. C2C12 myotubes were serum starved for 4 h prior to the treatment of DMSO (0.1%) as a vehicle control (CTRL) or AMPK activator 991 at the indicated concentration for 2 and 24 h. As negative and positive controls, C2C12 myotubes were treated with DMSO (0.1%, 24 h, lane 7) or CCCP (10 μM, 24 h, lane 8), respectively, without serum starvation. For ubiquitin pulldown, total lysates were incubated with ubiquitin‐binding resins derived from his‐halo‐ubiquilin1 UBA domain tetramer (UBA^UBQLN1^). Ubiquitin enriched extracts were analyzed by SDS‐PAGE and western blotting with the indicated antibodies. Representative images of n = 3 independent experiments are shown. Data are expressed as means ± SEM; *P < .05, **P < .01, ***P < .001, ****P < .0001 compared to CTRL. ^σ^ *P* < 0.05, ^δ^ *P* < 0.0001 compared to wild type 1 h. ^η^ *P* < 0.05, ^γ^ *P* < 0.0001 compared to wild type 2 h

AMPK activates ULK1 through direct phosphorylation at various sites.^39^, ^40^, ^41^ Importantly, ULK1 phosphorylation at Ser 555 regulates its activation and translocation to mitochondria.^39^, ^42^, ^43^, ^44^ Recent work in adipose tissue suggested a role for TBK1 and demonstrated that AICAR‐mediated AMPK activation‐induced TBK1 phosphorylation at Ser 172 via ULK1.^45^ Given that 1 hour of CCCP treatment activates both AMPK and TBK1 in C2C12 myotubes (Figures 2B and 4A), we hypothesized that the rapid activation of TBK1 may be AMPK dependent. Interestingly, we found that 991 treatment induces TBK1 phosphorylation at Ser 172, as well as ULK1 phosphorylation at Ser 555, in a dose dependent manner (Figure 7A). As expected, phosphorylation of AMPK and ACC were also increased following 991 treatment (Figure 7B).^38^ To demonstrate that TBK1 phosphorylation is AMPK dependent, we employed HEK293 AMPK α1/α2 deficient cells and found that TBK1 phosphorylation was attenuated following AMPK activation by 991 (Figure 7C). These data indicate that TBK1 phosphorylation is AMPK dependent. As expected, phosphorylation of ACC and ULK1 were increased in wild type, but dramatically attenuated in AMPK α1/α2 deficient cells following 991 treatment (Figure 7C). Finally, we demonstrated that AMPK‐mediated TBK1 phosphorylation in response to 991 treatment was independent of mitochondrial membrane depolarization and PINK1‐Parkin activity by showing that long OPA1 isoforms are not truncated, and an absence of Ub phosphorylation and CISD1 ubiquitylation (Figure 7D). Taken together, these data suggest that AMPK regulates the activation of TBK1 in skeletal muscle cells, and that this occurs in a PINK1‐Parkin independent manner.

## 4 DISCUSSION

The rationale for this study stemmed from our lack of current knowledge concerning the molecular mechanisms that underpin mitophagy in skeletal muscle. Much of the research that informs our understanding of the mechanisms governing mitophagy has been conducted in immortalized mammalian cells harboring systems that stably express non‐native PINK1 and/ or Parkin.^6^, ^7^, ^9^, ^14^, ^16^, ^19^, ^23^, ^24^, ^25^, ^26^ In skeletal muscle, our understanding of mitophagy, and how it changes in response to physiological stimuli such as exercise and aging, has been largely informed by the assessment of mitophagy markers,^46^, ^47^ rather than measuring mitophagy directly. Therefore, the main objective of this investigation was to directly measure mitophagy alongside its endogenous molecular mechanisms in skeletal muscle cells. The key questions we sought to answer were: (a) whether the PINK1‐Parkin signaling pathway is functionally active; (b) how AMPK is involved in the regulation of mitophagy; and (c) whether it cooperates with PINK1‐Parkin signaling. Our results demonstrate that the PINK1‐Parkin signaling pathway does operate in skeletal muscle cells, but its activation requires prolonged mitochondrial depolarization. We also demonstrate that AMPK activation stimulates mitochondrial fission via phosphorylation of MFF in skeletal muscle cells. Interestingly, in muscle cells, we found that AMPK activation stimulates TBK1 activation in a PINK1‐Parkin independent manner. To measure mitophagy directly, we harnessed the “mito‐QC” construct to generate a stable mitophagy reporter skeletal muscle cell line. This use of the tool allowed us to assess the occurrence of mitophagy and mitochondrial morphology in response to mitochondrial depolarization and AMPK activation.

The recent development of different tractable tools has made direct assessment of mitophagy in both cells and rodents feasible.^27^, ^32^, ^48^, ^49^, ^50^, ^51^ Here, we transfer a fluorescent, binary‐based mitophagy reporter construct previously developed by Allen et al^32^ into an immortalized mouse skeletal muscle (C2C12) cell line. After generating a C2C12 “mito‐QC” cell line capable of stably expressing the mitophagy reporter construct (mCherry‐GFP‐mtFIS1_101‐152_), we show that CCCP treatment for 24 hours significantly increases mitophagy by 18% in skeletal muscle cells (Figure 1). Unexpectedly, we observed no change in markers of mitochondrial protein content. Several studies have shown a marked reduction of mitochondrial content using microscopy and western blotting following treatment with CCCP.^24^, ^25^, ^26^ However, these investigations use cells that stably express Parkin which is thought to induce non‐native activation of its E3 ligase activity, particularly when fused with an exogenous tag at the N‐terminus of Parkin.^52^ Interestingly, Rakovic et al^53^ have shown Parkin overexpression to be required for a significant reduction in levels of mitochondrial proteins and mitochondrial mass following treatment with depolarizing agents, such as carbonyl cyanide‐4‐(trifluoromethoxy)phenylhydrazone (FCCP) or valinomycin. Our results are consistent with those of Rakovic et al,^53^ who also demonstrated that levels of mitochondrial proteins in neuroblastoma SH‐SY5Y cells expressing only endogenous Parkin to remain unchanged. However, unlike Rakovic et al,^53^ we observed a significant increase in mitophagy following treatment with CCCP (Figure 1B), albeit in the C2C12 mito‐QC skeletal muscle cell line developed here. In this instance, it is worth noting the importance of the methodological optimization required for the detection of mitophagy. For example, when piloting our experiments on glass coverslips, we noticed poor cell adherence, particularly upon drug treatment. Therefore, we suggest the use of imaging dishes with a tissue culture treated polymer for improved cell adherence during microscopy. Finally, the C2C12 “mito‐QC” muscle cell line we have established may serve as a useful tool for future studies to monitor mitophagy in skeletal muscle cells in real‐time. Moreover, given the recent finding that acute exercise induces mitophagy in mouse skeletal muscle,^2^ our C2C12 “mito‐QC” muscle cell line may help to explore this further by using electrical stimulation as a means to mimic muscle contraction.

Studying endogenous ubiquitylation events is challenging because of the lack of experimental tools sensitive enough for detection. Unlike protein phosphorylation where the use of phospho‐specific antibodies is common, similar tools for studying protein ubiquitylation are yet to be developed. Moreover, when using western blotting to study protein ubiquitylation, signal often appears diffuse and stretches from low to high molecular weight ranges making detection more difficult. Even though Ub is the substrate of PINK1 and Parkin is a Ub E3 ligase, to our knowledge, their intracellular activities are yet to be studied with endogenous levels of expression in skeletal muscle. To facilitate the study of endogenous PINK1‐Parkin signaling, we employed a specific Ub pull‐down technique. Using this technique, it was possible to unequivocally demonstrate the covalent attachment of poly‐Ub chains to the proteins of interest.^54^ First, we captured all the ubiquitylated proteins in cell lysates using Halo‐tagged TUBEs, which consist of tandem UBA domain repeats of the protein Ubiquilin‐1. After gel electrophoresis and protein transfer, membranes were probed with antibodies raised against our proteins of interest (Figures 3, 4C and 7D). This method allowed us to demonstrate endogenous PINK1‐Parkin signaling to be functional in skeletal muscle cells, despite their activation requiring prolonged (≥6 hours) CCCP treatment (Figure 3). In future, the application of this Ub pull‐down technique will not only be useful for studying endogenous PINK1‐Parkin activity, but could also be used to provide insight into ubiquitylation status in a wide range of tissues.

TBK1 is now known to be an important signaling node that operates as part of a positive feedback loop, helping to orchestrate efficient mitophagy through its association with autophagy receptors, such as OPTN, NDP52, and SQSTM1/ p62.^16^, ^17^, ^18^, ^19^, ^20^, ^21^ Although the role of TBK1 in mitophagy is subject to ongoing research, one of its identified functions is to enhance the binding capacity of OPTN with poly‐Ub chains.^19^ Thereafter, OPTN and other autophagy receptors are thought to link cargo to autophagosomal membranes via binding to Atg8 family proteins.^15^ Recent studies have suggested that TBK1 activation following phosphorylation at Ser 172^22^ requires both PINK1 and Parkin activity in response to mitochondrial depolarization.^16^, ^17^, ^23^ However, we found that under endogenous condition activation of TBK1 is independent of both PINK1 and Parkin activation (Figure 4B). This first became evident after we observed that TBK1 was phosphorylated (Figure 4A) prior to activation of PINK1 and Parkin following CCCP treatment (Figure 3). To verify this, we went on to demonstrate TBK1 phosphorylation following CCCP treatment was unchanged in both wild type and PINK1 KO HeLa cells (Figure 4B). Taken together, these data indicate that endogenous TBK1 is acutely activated following mitochondrial depolarization in a PINK1‐Parkin independent manner.

Mitochondrial fission plays a key role in mitophagy, preparing cells for the clearing of damaged mitochondrial fragments^30^ while preserving healthy mitochondria from the unchecked actions of the PINK1‐Parkin signaling pathway.^31^ It has recently been established that recruitment of the GTPase dynamin‐related protein 1 (DRP1) is essential for mitochondrial fission.^55^ MFF is a key receptor of DRP1^56^ and evidence suggests that AMPK directly phosphorylates MFF at Ser 146 in order to promote mitochondrial fission.^29^, ^30^ In accordance with previous research, we observed MFF phosphorylation following CCCP treatment (Figure 6B) similar to that of phosphorylated AMPK at Thr 172 and ACC at Ser 212 (Figure 2B). We also showed a robust increase in MFF phosphorylation at Ser 146 following 991 treatment in skeletal muscle cells. Using AMPK α1/α2 HEK293 deficient cells, we confirmed that MFF phosphorylation at Ser 146 is AMPK dependent (Figure 6C). In addition to this, we demonstrated the mitochondrial network to become more circular and less elongated following CCCP (Figure 1B) and 991 (Figure 5B) treatments, suggesting mitochondrial fission. Taken together, these data provide evidence that AMPK activation plays an important role in promoting mitochondrial fission in skeletal muscle cells by phosphorylating MFF.

To further probe the mechanistic basis through which AMPK initiates mitophagy, we treated mito‐QC skeletal muscle cells for 2 hours with 991 (Figure 5). Interestingly, we found that AMPK activation induces mitophagy on its own (Figure 5B) without the need for PINK1‐Parkin activity (Figure 7D). It is worth mentioning that after serum starvation, but prior to 991 treatment, we observed increases in red puncta, illustrating mitophagy in mito‐QC skeletal muscle cells (Figure 5A; CTRL). Unexpectedly, we observed a dose dependent increase in TBK1 activation following 991 treatment, suggesting that TBK1 phosphorylation may be mediated by AMPK. Moreover, phosphorylation of TBK1 increased across a 24 hours time course of CCCP treatment (Figure 4A), in parallel with ACC (Figure 2B) and MFF phosphorylation (Figure 6B). These data further support the hypothesis that TBK1 phosphorylation is controlled by AMPK. Finally, we confirm this hypothesis, by showing that TBK1 phosphorylation was dramatically reduced in HEK293 AMPK α1/α2 deficient cells. Previous research in adipose tissue has suggested that TBK1 is not a direct target of AMPK with ULK1 acting as an intermediate kinase responsible for TBK1 phosphorylation.^45^ In agreement with Zhao et al,^45^ we observed similar patterns of AMPK, ULK1, and TBK1 phosphorylation in wild type and AMPK α1/α2 deficient cells following 991 treatment, supporting the notion that AMPK‐dependent TBK1 phosphorylation may be mediated via ULK1.

**Figure 8:**
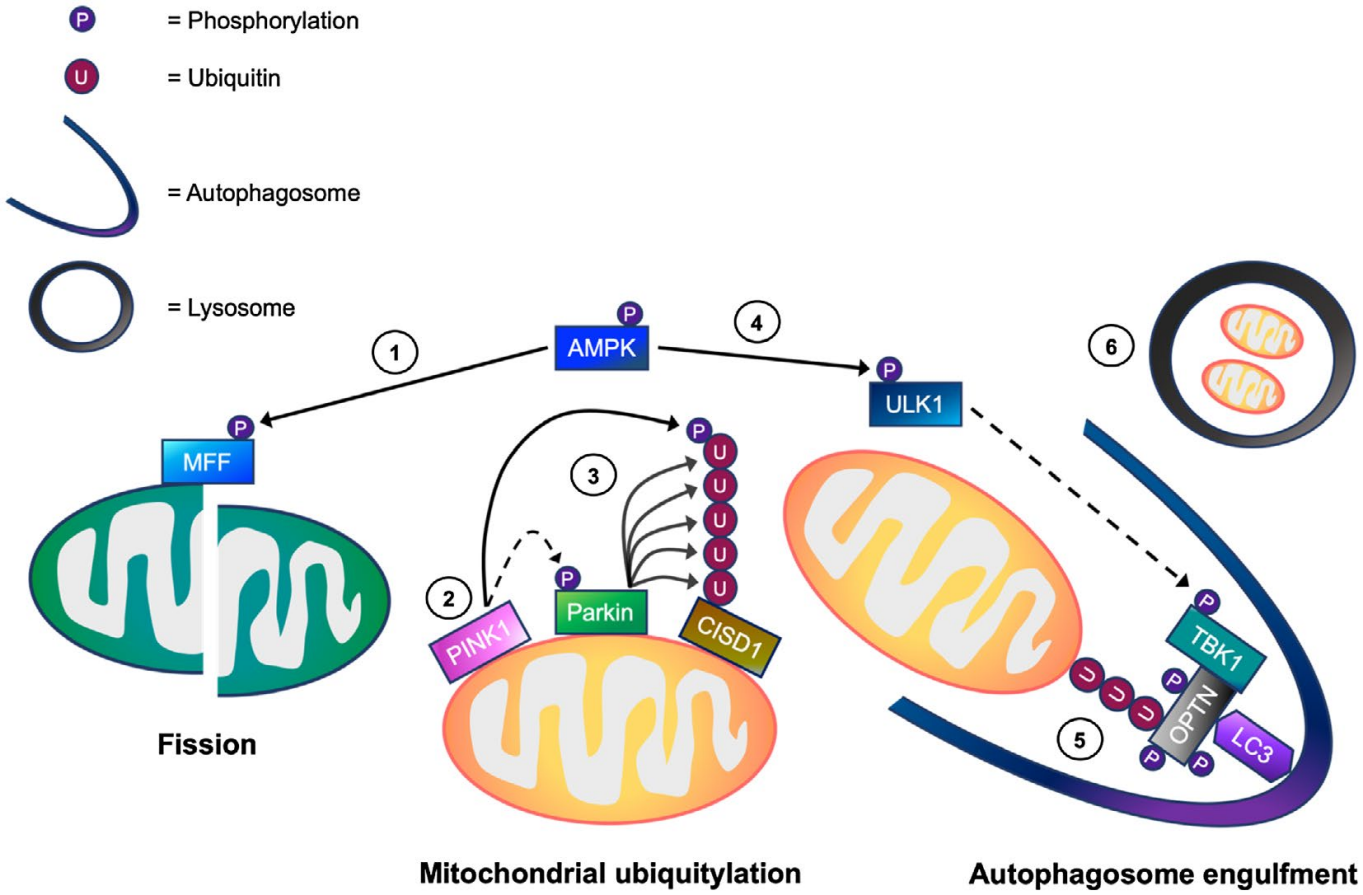
A working model of the mechanisms regulating mitophagy processing in skeletal muscle cells. In response to cellular energy stress, (1) AMP‐activated protein kinase (AMPK) activation initially promotes mitochondrial fission via direct phosphorylation of mitochondrial fission factor (MFF). This allows the separation of healthy and depolarized mitochondria. In depolarized mitochondria, (2) PTEN‐induced kinase 1 (PINK1) accumulates on the outer mitochondrial membrane (OMM), and phosphorylates both ubiquitin (Ub) and Parkin. (3) This is suggested to promote the recruitment of Parkin E3 ubiquitin ligase to the OMM. Parkin then ubiquitylates OMM proteins including CDGSH iron sulfur domain 1 (CISD1), facilitating mitochondrial ubiquitylation. Meanwhile, (4) AMPK activation leads to TBK1 phosphorylation possibly via ULK1, which in turn, is thought to translocate to the mitochondria. (5) The activation of TBK1 is proposed to enhance the binding capacity of autophagy receptors (eg, optineurin, OPTN) to ubiquitylated mitochondria, facilitating autophagosome engulfment. (6) Subsequently, the autophagosome fuses with the lysosome for mitochondrial degradation. → = Signaling thought to occur in skeletal muscle. ⇢ = Assumption based on signaling events in non‐muscle cell lines

Based on the findings in the present study and existing literature, we propose a working model of endogenous mechanisms that regulate mitophagy in skeletal muscle cells (Figure 8). In response to cellular energy stress, AMPK is activated and phosphorylates MFF, which in turn, functions as a receptor for DRP1‐mediated fission. Fission helps to separate healthy and depolarized mitochondria, with PINK1 and Parkin signaling being activated in the latter. First, PINK1 accumulates on the OMM and subsequently phosphorylates both the Ub, and the Ub ‐like domain of Parkin. This phosphorylation promotes the recruitment of Parkin to the OMM and helps to fully activate the Parkin Ub E3 ligase activity. Parkin then ubiquitylates a number of OMM proteins, such as CISD1, facilitating mitochondrial ubiquitylation. In contrast to previous research, we found the phosphorylation and activation of TBK1 was independent of both PINK1 and Parkin activity. Instead, our data suggests that TBK1 phosphorylation is mediated by AMPK, possibly via ULK1. The phosphorylation of TBK1 enhances the binding of autophagy receptors, such as OPTN, with poly‐Ub chains emanating from depolarized mitochondria. This promotes selective mitochondrial autophagy in skeletal muscle. In summary, our results support the hypothesis that AMPK drives mitophagy by enhancing mitochondrial fission and mitochondrial autophagosomal engulfment via TBK1 phosphorylation independently of PINK1 and Parkin. Thus, this study improves our understanding of the mechanisms that govern mitophagy in skeletal muscle.

## CONFLICT OF INTEREST

The authors declare no conflicts of interest.

## AUTHOR CONTRIBUTIONS

A.P. Seabright and Y.C. Lai conceived the study and designed experiments; A.P. Seabright, N.H.F. Fine, J.P. Barlow, S.O. Lord, I. Musa, and A. Gray performed experiments and analyzed the data; N.H.F. Fine, J.A. Bryant, M. Banzhaf, and D.J. Hodson provided microscopy support and expertise; A.P. Seabright and Y.C. Lai wrote the manuscript; M. Banzhaf, D.G. Hardie, G.G. Lavery, D.J. Hodson, and A. Philp helped to review the manuscript; all authors discussed the results and approved the final manuscript.

## Supporting information

Fig S1

Table S1
