# Supplementary material for: AMPK activation induces mitophagy and promotes mitochondrial fission while activating TBK1 in a PINK1‐Parkin independent manner
Source: FASEB J. 2020 Mar 22;34(5):6284–301. doi: 10.1096/fj.201903051R (PMC7212019; doi:10.1096/fj.201903051R)
Supplement: Supplementary file 1 — Fig S1 [file FSB2-34-6284-s002.pptx]

## Slide 1
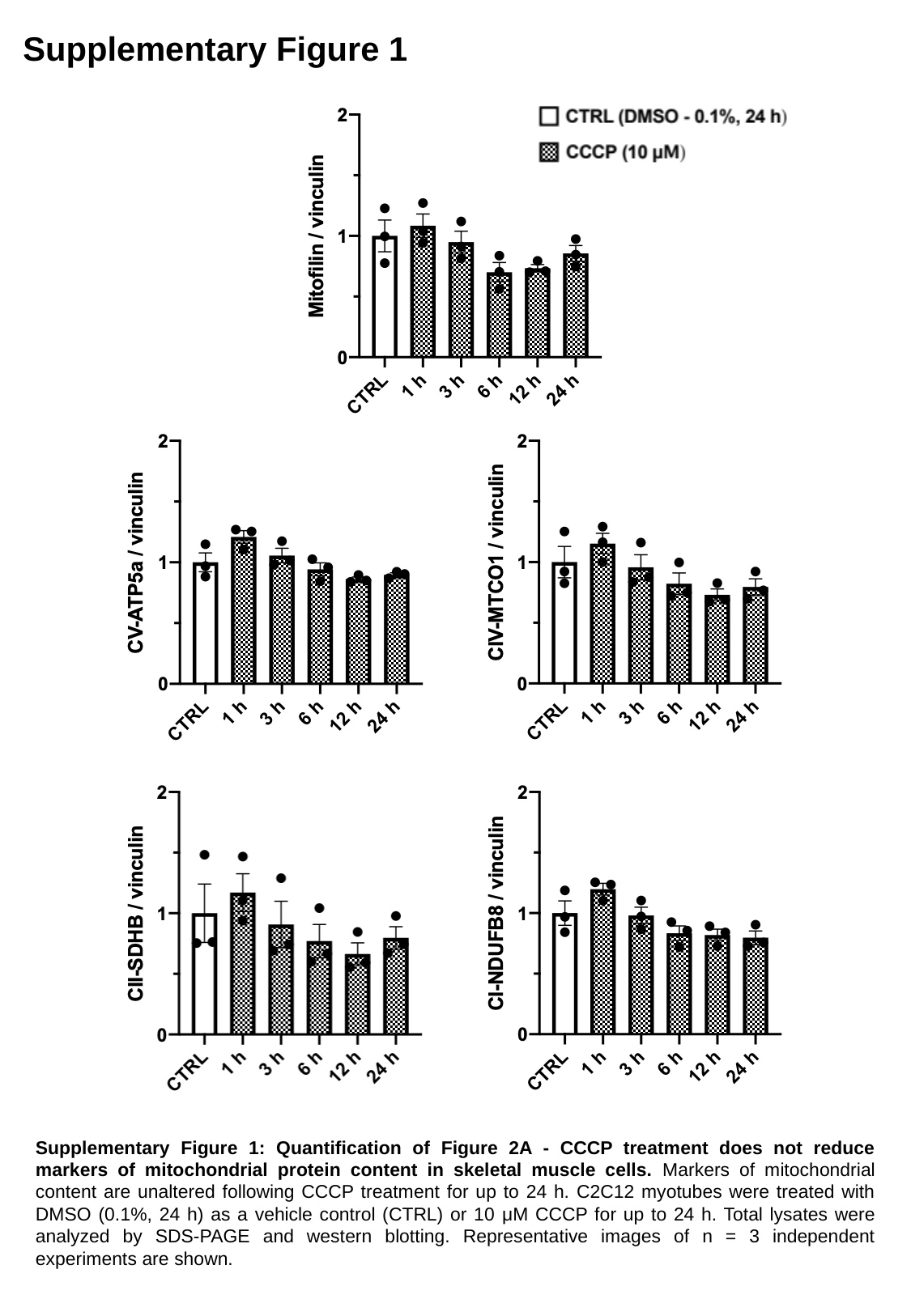

Supplementary Figure 1
Supplementary Figure 1: Quantification of Figure 2A - CCCP treatment does not reduce markers of mitochondrial protein content in skeletal muscle cells. Markers of mitochondrial content are unaltered following CCCP treatment for up to 24 h. C2C12 myotubes were treated with DMSO (0.1%, 24 h) as a vehicle control (CTRL) or 10 μM CCCP for up to 24 h. Total lysates were analyzed by SDS‐PAGE and western blotting. Representative images of n = 3 independent experiments are shown.
