# Supplementary material for: AMPK activation induces mitophagy and promotes mitochondrial fission while activating TBK1 in a PINK1‐Parkin independent manner
Source: FASEB J. 2020 Mar 22;34(5):6284–301. doi: 10.1096/fj.201903051R (PMC7212019; doi:10.1096/fj.201903051R)
Supplement: Supplementary file 2 — Table S1 [file FSB2-34-6284-s001.docx]

**Supplementary Table 1:** List of antibodies used.

| Supplementary Table 1 - Antibodies | | | |
| --- | --- | --- | --- |
| Antibody | **Source** | **Catalogue Number** | **Concentration** |
| ACC | Cell Signaling Technology | 3676 | 1:1000 |
| pSer79 ACC equivalent to mouse skeletal muscle ACC2 at Ser 212 | Cell Signaling Technology | 3661 | 1:1000 |
| AMPKα | Cell Signaling Technology | 2532 | 1:1000 |
| pThr172 AMPKα | Cell Signaling Technology | 2535 | 1:1000 |
| CISD1 | Proteintech | 16006-1-AP | 1:5000 |
| GAPDH | Cell Signaling Technology | 5174 | 1:5000 |
| Mitofilin | Proteintech | 10179-1-AP | 1:1000 |
| MFF | Cell Signaling Technology | 84580 | 1:5000 |
| pSer146 MFF equivalent to human at Ser 172 | Cell Signaling Technology | 49281 | 1:1000 |
| OPA1 | BD Biosciences | 612606 | 1:1000 |
| OXPHOS | Abcam | ab110413 | 1:1000 |
| TBK1 | Cell Signaling Technology | 3504 | 1:500 |
| PINK1 human residues 125–539 | Dundee Cell Products |  | 1:500 |
| pSer172 TBK1 | Cell Signaling Technology | 5483 | 1:500 |
| Ubiquitin | Biolegend | P4D1 | 1:2000 |
| pSer65 Ubiquitin | Sigma-Aldrich | ABS1513-I | 1:1000 |
| ULK1 | Cell Signaling Technology | 4773 | 1:1000 |
| pSer555 ULK1 | Cell Signaling Technology | 5869 | 1:1000 |
| Vinculin | Abcam | ab73412 | 1:2000 |
| Mouse IgG, HRP-linked | Cell Signaling Technology | 7076 | 1:10 000 |
| Rabbit IgG, HRP-linked | Cell Signaling Technology | 7074 | 1:10 000 |
